# Phytochemical Profiling and Biological Activities of Moroccan *Rhus pentaphylla*

**DOI:** 10.3390/molecules31203640

**Published:** 2026-10-12

**Authors:** Fatima-Ezzahra En-Naciri, Patrick Martin, Maryline Abert Vian, Nicolas Joly, Nihad Sahri, Asmae Alaoui

**Affiliations:** 1 Physico-Chemical Laboratory of Inorganic and Organic Materials (LPCMIO), Materials Science Center (MSC), École Normale Supérieure, Mohammed V University in Rabat, Rabat 10100, Morocco; 2 Chemistry Department, IUT de Béthune, Université d’Artois, 62408 Béthune, France; 3 GREEN Extraction Team, UMR 408, INRAE, Avignon University, 84914 Avignon, France

**Keywords:** *Rhus pentaphylla*, glycoconjugates, glycosylated metabolites, flavonoid glycosides, LC–ESI–MS/MS, antioxidant activity, enzyme inhibition, *Pseudomonas aeruginosa*, biofilm biomass

## Abstract

*Rhus pentaphylla* Desf., a medicinal plant traditionally used in North Africa, remains insufficiently investigated with respect to its glycoconjugate composition and biological properties. This study investigated the influence of extraction solvent on the LC–ESI–MS/MS profiles and biological activities of aqueous, ethanolic (EtOH_100_), and hydroethanolic (EtOH_50_) extracts of Moroccan *R. pentaphylla*, with particular emphasis on glycosylated metabolites. LC–ESI–MS/MS profiling resulted in the annotation of 39 metabolites belonging to several phytochemical classes, including phenolic acids, flavan-3-ols, coumarins, and numerous phenolic and flavonoid glycoconjugates. Citric acid, gallic acid, chlorogenic acid, esculetin, and caffeic acid were confirmed by comparison with in-house reference standards, whereas the remaining compounds were tentatively annotated based on their precursor ions and MS/MS fragmentation patterns through comparison with published literature and an in-house database. Several glycoconjugates, including caffeic acid glucoside, coumaric acid glycosides, and flavonoid glycosides, were tentatively annotated. Differences in the relative chromatographic responses of individual compounds were observed among the three extracts, highlighting the influence of extraction solvent on their phytochemical profiles. The hydroethanolic extract exhibited the highest total phenolic content and the strongest antioxidant activity (DPPH IC_50_ = 32.00 µg mL^−1^; ferric-reducing capacity = 81.56 mM FeSO_4_ equivalents g^−1^ extract), whereas the aqueous and ethanolic extracts showed greater tyrosinase inhibitory activity. All three extracts displayed comparable α-amylase inhibitory activity, with no statistically significant differences among them. Antibacterial activity was generally limited; however, the ethanolic extract produced the largest inhibition zone against *Pseudomonas aeruginosa* (14.01 ± 0.62 mm) and showed an MIC of 100 mg mL^−1^, whereas the MIC of the hydroethanolic extract was >100 mg mL^−1^. At the tested concentrations of 12.5 and 25 mg mL^−1^, the ethanolic extract significantly reduced *P. aeruginosa* biofilm biomass, whereas the hydroethanolic extract produced a significant reduction only at 25 mg mL^−1^. Both extracts significantly reduced the measured extracellular protein levels, while their effects on EPS-associated carbohydrates were more limited. Overall, extraction solvent influenced both the glycoconjugate-containing phytochemical profiles and the in vitro biological responses of *R. pentaphylla* extracts. The observed effects may reflect the combined contributions of multiple constituents rather than the activity of individual metabolites. Further bioassay-guided fractionation, structural confirmation, compound-specific quantitative analysis, and mechanistic investigations are required to identify the constituents contributing to these biological effects.

## 1. Introduction

Medicinal plants represent an important source of structurally diverse bioactive compounds and continue to contribute to the discovery of molecules with potential therapeutic applications. Among plant secondary metabolites, phenolic compounds, including phenolic acids, flavonoids, and tannins, have attracted considerable interest because of their broad spectrum of biological properties, particularly antioxidant, enzyme-modulatory, and antimicrobial activities [1,2,3]. These multifunctional properties have stimulated increasing interest in phenolic-rich plant species as potential sources of natural agents for applications related to metabolic disorders, skin-associated conditions, and microbial infections.

The genus *Rhus* (*Anacardiaceae*) comprises species recognized for their ethnomedicinal and phytochemical importance [1]. Among them, *Rhus pentaphylla* Desf. (syn. *Searsia pentaphylla*) is a xerophytic shrub occurring in Mediterranean and North African regions and has a long history of use in traditional medicine [4,5,6]. In Moroccan traditional medicine, different parts of the plant, including the leaves, roots, bark, and fruits, are used particularly for the treatment of gastrointestinal disorders and diarrhea [6]. Previous phytochemical investigations of *R. pentaphylla* and related *Rhus* species have revealed the presence of phenolic acids, flavonoids, tannins, and other secondary metabolites associated with diverse biological properties [1,4,5]. These ethnobotanical uses and the reported phytochemical richness of the species provide a rationale for further investigation of its aerial parts as a source of bioactive constituents.

The biological interest of phenolic-rich medicinal plants is closely related to their ability to act on multiple biochemical targets. Oxidative stress, resulting from an imbalance between reactive oxygen species production and antioxidant defenses, is involved in the development and progression of numerous chronic disorders. Plant-derived phenolic compounds may contribute to antioxidant defense through their radical-scavenging and reducing properties [2,3,7]. In addition, the modulation of key enzymes represents an important approach for the management of metabolic and skin-related conditions. Inhibition of α-amylase can reduce the enzymatic breakdown of dietary carbohydrates and thereby contribute to the control of postprandial hyperglycemia [8,9]. Tyrosinase, a key enzyme involved in melanin biosynthesis, is also an important target in the search for natural agents for the management of excessive pigmentation and for potential cosmetic applications. Several *Rhus* species have shown antioxidant and enzyme-inhibitory properties, supporting further investigation of their phenolic constituents as multifunctional bioactive agents [10].

In parallel with metabolic and oxidative-stress-related targets, the increasing prevalence of antimicrobial resistance has intensified the search for plant-derived compounds capable of modulating bacterial growth and pathogenicity. Species of the genus *Rhus* have demonstrated antibacterial activity against several clinically relevant microorganisms [11,12,13,14,15]. Among opportunistic pathogens, *Pseudomonas aeruginosa* is of particular interest because of its intrinsic resistance mechanisms and its ability to establish persistent infections through biofilm formation and the production of multiple virulence-associated factors [16,17]. Consequently, increasing attention has been directed toward strategies targeting biofilm formation and virulence-associated processes as complementary approaches to conventional growth-inhibitory antimicrobial treatments [16,17,18]. However, the effects of *R. pentaphylla* extracts on these bacterial traits remain insufficiently investigated.

Despite the ethnopharmacological relevance of *R. pentaphylla*, information integrating its phytochemical profile with multiple biological properties remains limited, particularly for Moroccan populations. Moreover, secondary metabolite profiles may vary among natural populations [19], while differences in solvent polarity may influence the recovery of phenolic and other secondary metabolites and, consequently, the biological responses of the resulting extracts [3]. Therefore, the present study aimed to compare aqueous, hydroethanolic, and ethanolic extracts of Moroccan *R. pentaphylla* aerial parts in terms of their extraction efficiency, phytochemical composition, and biological properties. Metabolite profiling was performed by LC–ESI–MS/MS, while the biological evaluation included antioxidant, α-amylase inhibitory, tyrosinase inhibitory, and antibacterial assays, together with the assessment of biofilm formation, extracellular protein production, and exopolysaccharide production in *P. aeruginosa*. By integrating phytochemical profiling with these complementary biological assays, this study sought to provide a broader understanding of the influence of extraction solvent on the phytochemical and functional properties of *R. pentaphylla* and to provide a basis for further investigation of this medicinal species as a potential source of bioactive compounds.

## 2. Results

### 2.1. Phytochemical Composition and Extraction Yield

The extraction yields and the total phenolic (TPC), total flavonoid (TFC), total protein (TP), and total sugar (TS) contents of the three *R. pentaphylla* extracts are presented in Table 1. Extraction yield varied according to the solvent system, with the hydroethanolic extract (EtOH_50_) showing the highest yield (37.95%), followed by the ethanolic extract (EtOH_100_; 24.50%) and the aqueous extract (6.77%). EtOH_50_ also exhibited the highest TPC (161.05 ± 0.011 mg GAE g^−1^ extract), whereas EtOH_100_ showed the highest TFC (66.50 ± 0.005 mg QE g^−1^ extract) and TP (90.19 ± 0.013 mg BSAE g^−1^ extract). The aqueous extract showed the highest TS value (144.82 ± 0.016 mg GE g^−1^ extract). These results show that solvent composition differentially affected extraction yield and the recovery of the measured phytochemical classes.

**Figure 1:**
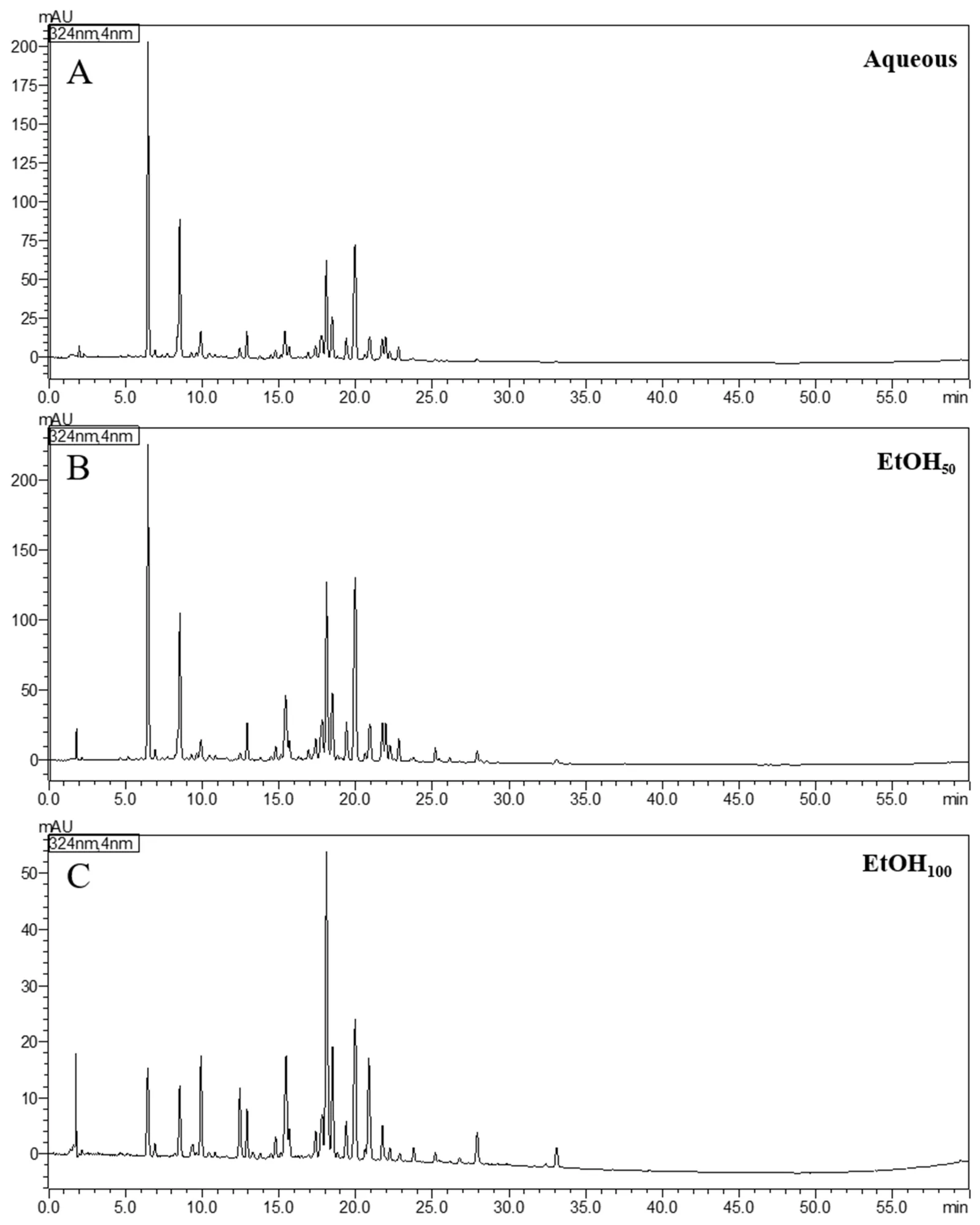
LC-ESI-MS/MS chromatograms of *R. pentaphylla* extracts: (**A**) aqueous extract; (**B**) hydroethanolic extract (EtOH_50_); and (**C**) ethanolic extract (EtOH_100_).

LC–ESI–MS/MS analysis resulted in the annotation of 39 metabolites belonging to several phytochemical classes, including phenolic acids, flavan-3-ols, coumarins, and flavonoid glycosides (Table 2). Citric acid, gallic acid, chlorogenic acid, esculetin, and caffeic acid were confirmed by comparison with in-house reference standards, whereas the remaining compounds were tentatively annotated based on their molecular ions and MS/MS fragmentation patterns through comparison with published literature and an in-house database (Figure 1).

The three *R. pentaphylla* extracts showed distinct metabolite profiles, with differences in the relative chromatographic responses of individual compounds among the aqueous, hydroethanolic, and ethanolic extracts. Several glycosylated phenolic compounds, including caffeic acid glucoside, coumaric acid glycosides, and flavonol glycosides, were tentatively annotated. The relative peak areas of these compounds varied among the three extracts.

Among the hydroxycinnamic acid derivatives, chlorogenic acid showed a higher relative chromatographic response in the aqueous and hydroethanolic extracts than in the ethanolic extract. A caffeoylquinic acid isomer tentatively annotated as cryptochlorogenic acid showed a similar response pattern. Other hydroxycinnamic acid derivatives included caffeic acid, which was confirmed using an in-house reference standard, as well as caffeic acid glucoside, p-coumaroylquinic acid, coumaric acid, and coumaric acid glycosides, which were tentatively annotated. Some tentatively annotated coumaric acid derivatives, together with standard-confirmed esculetin, showed higher relative chromatographic responses in the ethanolic extract than in the aqueous and hydroethanolic extracts.

Overall, the LC–ESI–MS/MS profiles revealed qualitative differences and variations in the relative chromatographic response of individual compounds among the three extracts. These relative responses are based on chromatographic peak areas and should not be interpreted as absolute concentrations or used to compare the abundance of different compounds.

### 2.2. Antioxidant Activity

The antioxidant properties of the *R. pentaphylla* extracts were evaluated using the DPPH radical-scavenging and ferric-reducing capacity assays (Table 3). Among the three extracts, the hydroethanolic extract (EtOH_50_) showed the highest DPPH radical-scavenging activity, with the lowest IC_50_ value (32.00 ± 0.005 µg mL^−1^), followed by the ethanolic extract (EtOH_100_; 39.60 ± 0.019 µg mL^−1^) and the aqueous extract (87.01 ± 0.005 µg mL^−1^). Ascorbic acid, used as the reference antioxidant, exhibited an IC_50_ value of 3.60 ± 0.003 µg mL^−1^.

In the ferric-reducing capacity assay, EtOH_50_ also showed the highest value (81.56 ± 0.069 mM FeSO_4_ equivalents g^−1^ extract), followed by EtOH_100_ (70.44 ± 0.059 mM FeSO_4_ equivalents g^−1^ extract) and the aqueous extract (66.57 ± 0.012 mM FeSO_4_ equivalents g^−1^ extract). Quercetin, used as the reference antioxidant for this assay, showed a ferric-reducing capacity of 57.48 ± 0.55 mM FeSO_4_ equivalents g^−1^ extract.

### 2.3. Enzyme Inhibitory Activities

Table 4 summarizes the α-amylase and tyrosinase inhibitory activities of the aqueous, hydroethanolic (EtOH_50_), and ethanolic (EtOH_100_) extracts of *R. pentaphylla*. For α-amylase inhibition, the aqueous extract showed the lowest IC_50_ value (1.25 ± 0.08 mg mL^−1^), followed by EtOH_50_ (1.49 ± 0.09 mg mL^−1^) and EtOH_100_ (1.60 ± 0.12 mg mL^−1^). However, one-way ANOVA revealed no statistically significant differences among the three extracts (*p* = 0.0968). All three extracts showed higher IC_50_ values than the reference inhibitor acarbose (0.61 ± 0.03 mg mL^−1^). In contrast, significant differences were observed among the extracts for tyrosinase inhibition (*p* = 0.0091). The aqueous and EtOH_100_ extracts showed comparable inhibitory activities, with IC_50_ values of 0.27 ± 0.01 and 0.27 ± 0.05 mg mL^−1^, respectively, and both exhibited significantly lower IC_50_ values than EtOH_50_ (0.42 ± 0.04 mg mL^−1^). Kojic acid, used as the reference inhibitor, showed an IC_50_ value of 0.06 ± 0.05 mg mL^−1^.

### 2.4. Antibacterial Activity

The antibacterial activity of *R. pentaphylla* extracts was evaluated against five bacterial strains using agar disc diffusion as an initial screening method. Inhibition zone diameters were measured to compare the effects of the aqueous, hydroethanolic (EtOH_50_), and ethanolic (EtOH_100_) extracts. Based on the disc diffusion results, EtOH_50_ and EtOH_100_ were subsequently selected for MIC determination against *P. aeruginosa* using the broth microdilution method. The antibacterial results are summarized in Table 5.

As shown in Table 5, EtOH_100_ produced the largest inhibition zone against *Pseudomonas aeruginosa* (14.01 ± 0.62 mm), whereas the aqueous and EtOH_50_ extracts produced inhibition zones of 6.82 ± 0.05 and 7.44 ± 0.01 mm, respectively. Ampicillin produced an inhibition zone of 15.01 ± 0.03 mm against the same strain. For *Escherichia coli*, *Enterococcus faecalis*, *Staphylococcus aureus*, and *Salmonella typhi*, all extracts produced inhibition zone diameters ranging from 6.30 to 6.52 mm. Given that the reported diameters include the 6 mm paper disc, these values indicate minimal inhibition beyond the disc margin. In contrast, ampicillin produced substantially larger inhibition zones against all tested bacterial strains. Overall, among the tested extract–strain combinations, EtOH_100_ showed the most pronounced inhibitory effect against *P. aeruginosa* in the disc diffusion assay.

Based on the preliminary disc diffusion screening, EtOH_100_ and EtOH_50_ were selected for further evaluation against *P. aeruginosa* using the broth microdilution method (Table 6). Due to extract solubility limitations, 100 mg mL^−1^ was the highest concentration tested. EtOH_100_ completely inhibited visible bacterial growth at 100 mg mL^−1^ and was therefore assigned an MIC of 100 mg mL^−1^. At this concentration, spectrophotometric analysis showed 98.25 ± 0.01% growth inhibition. In contrast, EtOH_50_ produced 81.56 ± 0.07% growth inhibition at 100 mg mL^−1^ but did not completely suppress visible bacterial growth. Consequently, its MIC was not reached within the tested concentration range and was reported as >100 mg mL^−1^. Ampicillin showed an MIC of 0.031 mg mL^−1^.

### 2.5. Anti-Biofilm Activity and Associated Extracellular Factors

**Figure 2:**
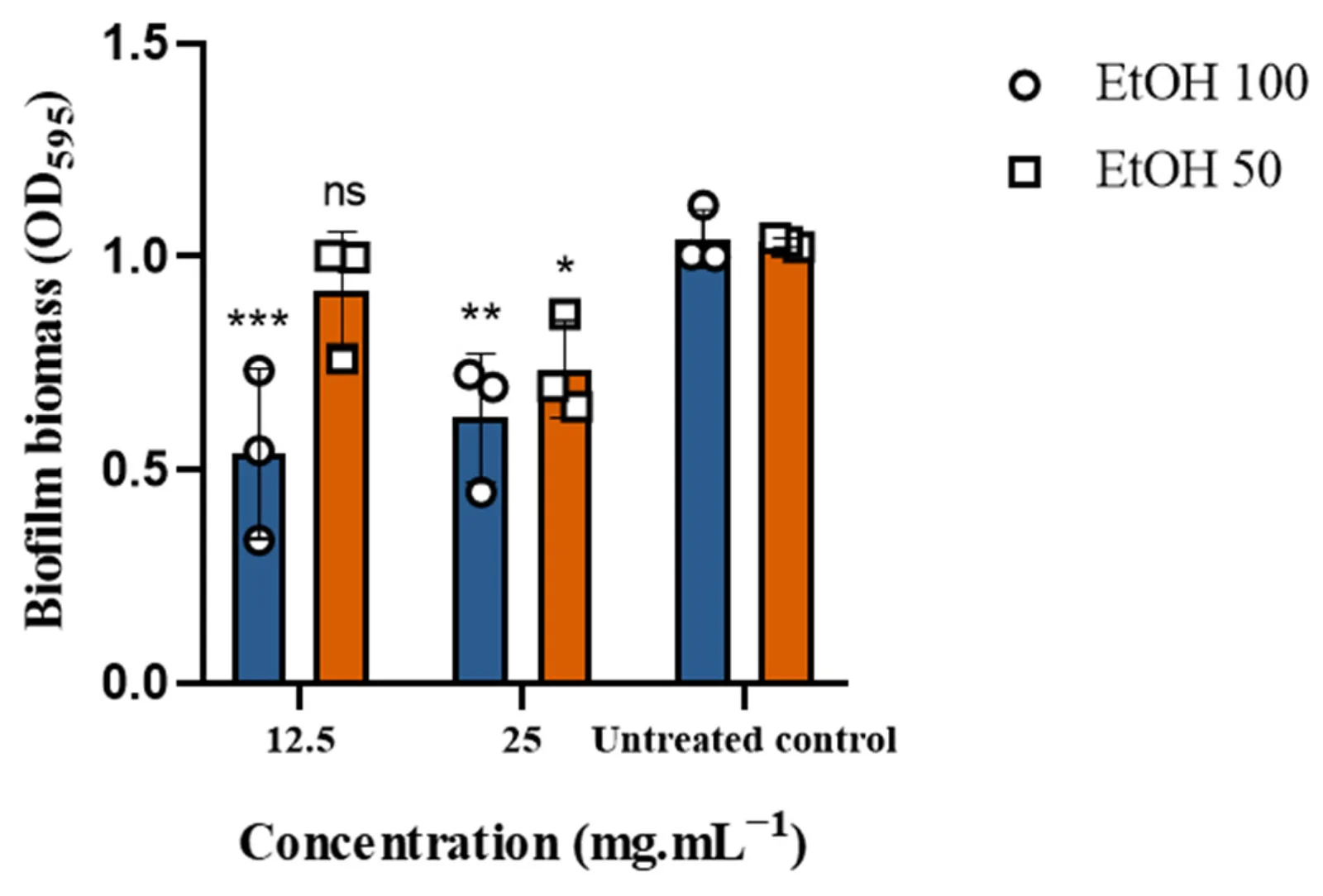
Effect of *R. pentaphylla* extracts on biofilm biomass in *Pseudomonas aeruginosa*. EtOH_100_ and EtOH_50_ were tested at concentrations of 12.5 and 25 mg mL^−1^. Data are expressed as the mean ± SD (*n* = 3 independent biological experiments), with individual data points shown. Statistical significance was assessed by two-way ANOVA followed by Dunnett’s multiple-comparisons test against the untreated control. * *p* < 0.05, ** *p* < 0.01, *** *p* < 0.001; ns, not significant (*p* ≥ 0.05).

Biofilm formation is an important characteristic of *P. aeruginosa* that contributes to its persistence and reduced susceptibility to antimicrobial treatment. The effects of the *R. pentaphylla* extracts on biofilm biomass were therefore evaluated at concentrations of 12.5 and 25 mg mL^−1^. As shown in Figure 2, biofilm biomass was significantly affected by treatment concentration (two-way ANOVA, *p* = 0.0008) and extraction solvent (*p* = 0.0201), whereas the interaction between these factors was not statistically significant (*p* = 0.0587).

Dunnett’s multiple-comparisons test showed that the ethanolic extract significantly reduced biofilm biomass by 48.37% at 12.5 mg mL^−1^ (*p* = 0.0008) and by 40.31% at 25 mg mL^−1^ (*p* = 0.0033), compared with the untreated control. The hydroethanolic extract reduced biofilm biomass by 9.97% and 28.75% at 12.5 and 25 mg mL^−1^, respectively. The reduction observed at 25 mg mL^−1^ was statistically significant (*p* = 0.0278), whereas that observed at 12.5 mg mL^−1^ was not significant (*p* = 0.4756).

These findings demonstrate a solvent- and concentration-dependent reduction in biofilm biomass under the experimental conditions. However, because the measurements were not normalized to viable cell counts or total bacterial biomass, the observed reductions cannot be interpreted as evidence of a specific antibiofilm or antivirulence mechanism.

In addition to biofilm biomass, the effects of *R. pentaphylla* extracts on extracellular matrix-associated components of *P. aeruginosa* were evaluated by measuring total extracellular protein and EPS-associated carbohydrate levels at 12.5 and 25 mg mL^−1^. The results were compared with those of the untreated control and azithromycin (2 µg mL^−1^), which was used as the reference treatment.

**Figure 3:**
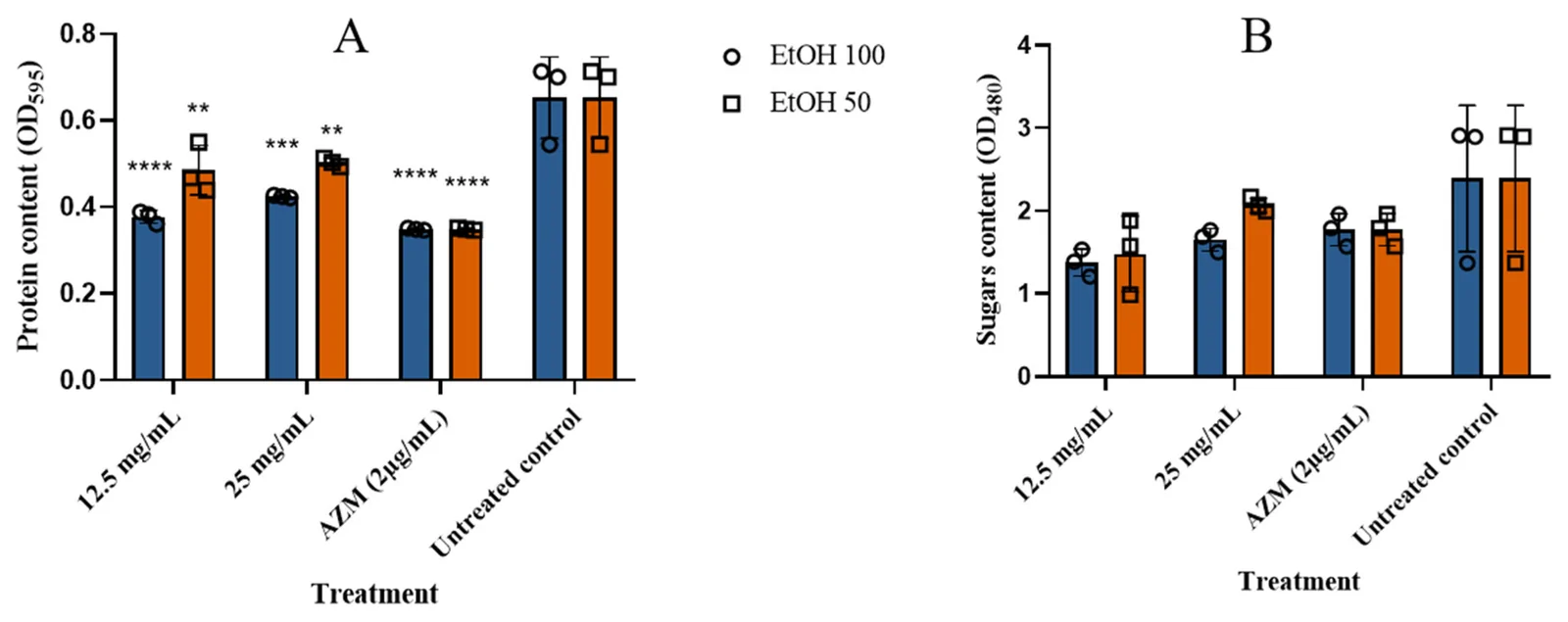
Effect of *R. pentaphylla* extracts on *P. aeruginosa* extracellular matrix. (**A**) Extracellular protein content (OD_595_) and (**B**) exopolysaccharide (EPS) content (OD_480_) at sub-inhibitory concentrations and azithromycin (AZM) relative to untreated control (0 MIC). Data represent mean ± SD (*n* = 3 independent biological replicates shown as individual points). Statistical significance was assessed by two-way ANOVA with Dunnett’s post hoc test against 0 MIC. ** *p* < 0.01, *** *p* < 0.001, **** *p* < 0.0001. Bars without asterisks indicate no significant difference (*p* ≥ 0.05).

For total extracellular protein (Figure 3A), two-way ANOVA revealed significant effects of treatment concentration (*p* < 0.0001) and extract type (*p* = 0.0417). Dunnett’s multiple-comparisons test showed significant reductions relative to the untreated control for EtOH_100_ at 12.5 mg mL^−1^ (*p* < 0.0001) and 25 mg mL^−1^ (*p* = 0.0002). EtOH_50_ also significantly reduced total extracellular protein levels at 12.5 mg mL^−1^ (*p* = 0.0030) and 25 mg mL^−1^ (*p* = 0.0072). Azithromycin similarly produced a significant reduction in total extracellular protein levels (*p* < 0.0001).

For EPS-associated carbohydrates (Figure 3B), two-way ANOVA showed a significant overall effect of treatment concentration (*p* = 0.0267), whereas the effect of extract type was not statistically significant (*p* = 0.5208). Dunnett’s multiple-comparisons test against the untreated control showed that the reductions observed at 12.5 mg mL^−1^ did not reach statistical significance for either EtOH_100_ (*p* = 0.0547) or EtOH_50_ (*p* = 0.0899). Similarly, no statistically significant differences were observed at 25 mg mL^−1^ (*p* > 0.05). Overall, the extracts significantly affected total extracellular protein levels, whereas their effects on EPS-associated carbohydrates were more limited and did not reach statistical significance in the individual comparisons with the untreated control.

## 3. Discussion

Oxidative stress is a common underlying factor in several pathological processes and has therefore become an important target in the investigation of plant-derived bioactive compounds [20,21,22,23,24,25]. In this context, the antioxidant properties of *R. pentaphylla* extracts are of particular interest given the diversity of phenolic constituents detected in the present study. The three extracts displayed distinct antioxidant profiles, with the hydroethanolic extract showing the strongest activity in both the DPPH and ferric-reducing capacity assays, as well as the highest total phenolic content. This pattern suggests that the extraction solvent influenced both the overall recovery of phenolic compounds and the composition of the resulting extracts, which may have contributed to the observed differences in antioxidant responses. Similar solvent-dependent relationships between phenolic composition and antioxidant activity have been reported for other plant extracts [3,26,27,28,29]. Phenolic compounds, including hydroxycinnamic acids and flavonoids, may contribute to antioxidant activity through their ability to interact with reactive species and participate in redox-related processes [30,31,32]. However, the differences observed among the extracts indicate that antioxidant capacity cannot necessarily be attributed to total phenolic content alone. Variations in qualitative composition, together with possible additive, synergistic, or antagonistic interactions among extract constituents, may also influence the measured activity [26,27,28,29,33]. LC–ESI–MS/MS profiling further highlighted the chemical complexity of the extracts. Citric acid, gallic acid, chlorogenic acid, esculetin, and caffeic acid were confirmed by comparison with in-house reference standards. The remaining compounds, comprising phenolic acids, flavan-3-ols, coumarin-related compounds, and flavonoid glycosides, were tentatively annotated through comparison with published literature and an in-house database. Chlorogenic acid showed a higher relative chromatographic response in the aqueous and hydroethanolic extracts than in the ethanolic extract. A caffeoylquinic acid isomer tentatively annotated as cryptochlorogenic acid showed a similar response pattern. Among these, caffeic acid glucoside, coumaric acid derivatives, and several glycosylated flavonoids showed different relative chromatographic responses among the three extracts. These differences may reflect the influence of solvent polarity on the extraction of compounds with different physicochemical properties. The presence of chlorogenic acid and other hydroxycinnamic acid derivatives is of particular interest because this class of compounds has been widely investigated for its antioxidant and other biological properties [34,35,36]. Nevertheless, except for the five compounds confirmed using in-house reference standards, the LC–ESI–MS/MS assignments remain tentative. Moreover, the detected or tentatively annotated metabolites should be regarded as components of the overall phytochemical profile rather than as individually confirmed determinants of the observed antioxidant effects. Isolation, compound-specific quantification, and individual biological evaluation would be required to establish their respective contributions.

The antioxidant findings may also be relevant to the investigation of the potential metabolic applications of *R. pentaphylla*. Oxidative imbalance is associated with metabolic disorders, including diabetes, in which excessive generation of reactive oxygen species may contribute to cellular and tissue damage [22,37,38].

Inhibition of carbohydrate-hydrolyzing enzymes such as α-amylase is considered one approach for limiting the rapid release of glucose following carbohydrate digestion. In the present study, all three *R. pentaphylla* extracts inhibited α-amylase, although their IC_50_ values were higher than those of the reference inhibitor acarbose. No statistically significant differences were observed among the three extracts (*p* = 0.0968), indicating comparable α-amylase inhibitory activity under the experimental conditions used. Previous investigations of *Rhus* species have similarly reported α-amylase inhibitory effects, supporting further investigation of phenolic-rich extracts in this context [8,39]. However, the activity observed in the present study cannot be attributed to specific metabolites, as the extracts were tested as complex mixtures and their individual constituents were not evaluated independently.

The enzymatic evaluation also included tyrosinase inhibition, which is of interest because tyrosinase plays a key role in melanin biosynthesis and represents an important target in pigmentation-related and cosmetic research. In contrast to the α-amylase results, significant differences in tyrosinase inhibition were observed among the extracts (*p* = 0.0091). The aqueous and EtOH_100_ extracts showed comparable inhibitory activities, with lower IC_50_ values than EtOH_50_. Interestingly, this pattern differed from that observed for antioxidant activity and total phenolic content, for which EtOH_50_ showed the highest values. This finding suggests that tyrosinase inhibition may depend more on the qualitative composition and relative distribution of specific constituents than on total phenolic content alone. Phenolic compounds, including flavonoids and hydroxycinnamic acid derivatives, have previously been investigated for their interactions with tyrosinase and their effects on melanogenesis-related processes [40,41,42,43,44]. Nevertheless, further fractionation and compound-specific evaluation would be required to identify the constituents primarily associated with the observed tyrosinase inhibitory activity.

The coexistence of antioxidant and tyrosinase-inhibitory activities in *R. pentaphylla* extracts suggests that their potential applications may extend beyond metabolic targets to pigmentation-related and cosmetic research. However, as with the antioxidant and α-amylase assays, the present results do not establish that any single metabolite is responsible for the observed activities.

The increasing emergence of antibiotic-resistant pathogens has stimulated interest in alternative antimicrobial strategies, including approaches targeting bacterial phenotypes associated with persistence and colonization rather than relying exclusively on bacterial killing. *P. aeruginosa* is a clinically relevant opportunistic pathogen characterized by intrinsic resistance mechanisms and a pronounced capacity for biofilm formation. In the present study, the antibacterial effects of *R. pentaphylla* extracts varied according to the bacterial strain and extraction solvent. EtOH_100_ produced the largest inhibition zone against *P. aeruginosa* (14.01 ± 0.62 mm), whereas the aqueous and EtOH_50_ extracts produced substantially smaller inhibition zones. In contrast, inhibition zone diameters against *E. coli*, *E. faecalis*, *S. aureus*, and *S. typhi* ranged from 6.30 to 6.52 mm. Given that these diameters include the 6 mm paper disc, the latter values indicate minimal inhibition beyond the disc margin.

Differences in bacterial susceptibility to plant extracts may be influenced by several factors, including cell-envelope architecture, membrane permeability, intrinsic resistance mechanisms, and the physicochemical properties of extract constituents [45,46]. Previous studies on *R. pentaphylla* have likewise reported variable antibacterial activity depending on the extraction procedure, solvent polarity, and microorganisms tested [47,48,49]. The agar diffusion results should nevertheless be interpreted cautiously, as inhibition-zone diameters depend not only on antibacterial effects but also on the ability of extract constituents to diffuse through the agar matrix. This consideration is particularly relevant for chemically complex crude plant extracts. Accordingly, inhibition-zone diameters should not be regarded as a direct measure of intrinsic antibacterial potency. Ampicillin produced markedly larger inhibition zones against the tested strains and served as the reference antibacterial agent.

Broth microdilution further clarified the activity of EtOH_100_ and EtOH_50_ against *P. aeruginosa*. Due to extract solubility limitations, 100 mg mL^−1^ was the highest concentration tested. EtOH_100_ completely inhibited visible bacterial growth at this concentration and was therefore assigned an MIC of 100 mg mL^−1^. Spectrophotometric analysis at the same concentration showed 98.25 ± 0.01% growth inhibition. In contrast, EtOH_50_ produced 81.56 ± 0.07% growth inhibition at 100 mg mL^−1^ but did not completely suppress visible bacterial growth; consequently, its MIC was not reached within the tested concentration range and was reported as >100 mg mL^−1^. Ampicillin exhibited an MIC of 0.031 mg mL^−1^, demonstrating substantially greater antibacterial activity against *P. aeruginosa* under the experimental conditions used.

The relatively high concentrations required for antibacterial activity are consistent with the behavior of many crude plant extracts, whose effects may reflect the combined contribution of multiple constituents rather than the potency of a single purified antimicrobial compound [50]. The differences observed between EtOH_100_ and EtOH_50_ may therefore be related to variations in their phytochemical composition and relative distribution of constituent metabolites. Beyond direct growth inhibition, biofilm formation represents an important feature of *P. aeruginosa* that contributes to persistence and reduced susceptibility to antimicrobial treatment. Accordingly, EtOH_100_ and EtOH_50_ were further evaluated at 12.5 and 25 mg mL^−1^ for their effects on biofilm biomass and extracellular matrix-associated components. EtOH_100_ significantly reduced biofilm biomass at both concentrations, whereas EtOH_50_ produced a significant reduction only at 25 mg mL^−1^. Notably, the effect of EtOH_100_ was not concentration dependent within the tested range, as biofilm biomass reduction was greater at 12.5 mg mL^−1^ (48.37%) than at 25 mg mL^−1^ (40.31%). Both extracts also significantly reduced total extracellular protein levels, whereas their effects on EPS-associated carbohydrates were more limited and did not reach statistical significance in the individual comparisons with the untreated control.

These findings should be interpreted within the scope of the parameters actually measured. Total extracellular protein and EPS-associated carbohydrate levels were quantified as collective indicators of extracellular matrix-associated components, whereas individual proteins, polysaccharides, or specific virulence factors were not characterized. Therefore, the observed changes should not be interpreted as evidence of direct inhibition of specific virulence mechanisms. Moreover, without normalization to bacterial biomass or viable cell counts under the same treatment conditions, it cannot be established whether the observed effects on biofilm biomass and extracellular components occurred independently of changes in bacterial growth. Further studies incorporating appropriate growth normalization, together with targeted analysis of individual matrix and virulence-associated components, would be required to clarify the mechanisms underlying these effects.

The observed biofilm-associated effects may nevertheless be considered in relation to the complex phytochemical composition of the extracts. Flavonoids and phenolic acids have previously been investigated for their effects on bacterial communication, biofilm development, and extracellular processes in *P. aeruginosa* [51,52]. Quercetin, for example, has been reported to affect quorum-sensing-related processes, biofilm formation, and the production of bacterial extracellular factors [51]. However, the quercetin glucuronide tentatively annotated in the present study cannot be considered individually responsible for the observed effects because it was neither isolated nor tested independently. Similarly, chlorogenic acid has previously been associated with antibacterial effects, including alterations in bacterial membrane integrity and cellular function [53]. Whether chlorogenic acid contributed to the biofilm-associated effects observed here cannot, however, be determined from the present data. The measured responses may instead reflect the combined contribution of multiple phenolic and non-phenolic constituents, although potential additive or synergistic interactions would require direct experimental confirmation.

Taken together, the results indicate that solvent composition influenced both the phytochemical profiles and the biological properties of the resulting *R. pentaphylla* extracts. No single extract exhibited the same pattern of activity across all evaluated endpoints. EtOH_50_ showed the highest antioxidant activity and total phenolic content, whereas the aqueous and EtOH_100_ extracts exhibited greater tyrosinase inhibitory activity than EtOH_50_. Differences among the extracts were also observed in their antibacterial and biofilm-associated effects. These variations may reflect differences in the qualitative composition and relative MS responses of metabolites detected in the extracts. However, the multifunctional behavior of these chemically complex extracts should not be interpreted as evidence that one or a few major metabolites account for the observed biological activities. Rather, multiple constituents may contribute to the measured responses, and their respective contributions remain to be established experimentally.

The present findings should be interpreted within the exploratory and in vitro scope of the study. Although citric acid, gallic acid, chlorogenic acid, esculetin, and caffeic acid were confirmed by comparison with in-house reference standards, the remaining LC–ESI–MS/MS assignments are tentative. Moreover, the extracts represent chemically complex mixtures, and no individual metabolite was isolated, quantified using a compound-specific calibration curve, or evaluated independently. Consequently, associations between particular metabolites and the observed antioxidant, enzyme-inhibitory, antibacterial, or biofilm-associated effects cannot be interpreted as evidence of direct causation. Bioassay-guided fractionation, followed by the isolation, structural confirmation, compound-specific quantification, and individual biological evaluation of candidate metabolites, would be required to determine the contribution of individual constituents. Combination-based testing could subsequently be used to investigate potential additive, synergistic, or antagonistic interactions among these constituents. Quantitative analysis using appropriate analytical standards and compound-specific calibration curves would also help establish relationships between metabolite concentrations and biological responses. In addition, normalization to bacterial biomass or viable cell counts, together with targeted analysis of specific extracellular matrix and virulence-associated components, would strengthen the interpretation of the biofilm-related findings. Finally, mechanistic and in vivo studies will be required to assess the biological relevance, safety, and potential therapeutic or cosmeceutical applications of *R. pentaphylla* extracts.

## 4. Materials and Methods

### 4.1. Sample Preparation and Extraction

#### 4.1.1. Plant Material

The aerial parts of *Rhus pentaphylla* Desf. were collected in October 2023 from several individual plants belonging to a single population at Douar Oued Baht, approximately 15 km from Khemisset, Morocco. Botanical identification was initially performed by Prof. Rouane (École Normale Supérieure, Rabat, Morocco) and subsequently confirmed by Prof. Khamar (Department of Botany, Scientific Institute of Rabat, Rabat, Morocco). A voucher specimen (RAB114123) was deposited in the herbarium of the Scientific Institute of Rabat. The collected plant material was air-dried at room temperature in the dark for 10 days and then ground to a fine powder using a GM200 laboratory grinder (Retsch, GmbH, Haan, Germany). The powdered material was stored in airtight containers at room temperature until extraction. The exact GPS coordinates and the phenological stage of the plants were not formally recorded at the time of collection and were therefore not retrospectively assigned.

#### 4.1.2. Ultrasound-Assisted Extraction

Ultrasound-assisted extraction (UAE) was selected to enhance solvent penetration and mass transfer under mild extraction conditions. Distilled water, absolute ethanol, and 50:50 (*v*/*v*) ethanol–water were selected to cover different solvent polarities and facilitate the recovery of phytochemicals with different physicochemical properties. The extracts obtained using these solvents are hereafter referred to as the aqueous, ethanolic (EtOH_100_), and hydroethanolic (EtOH_50_) extracts, respectively.

For each extraction, 10 g of powdered aerial parts were mixed with 100 mL of the corresponding extraction solvent, corresponding to a solid-to-solvent ratio of 1:10 (*w*/*v*). The suspensions were sonicated in an ultrasonic bath (Elmasonic, Elma Schmidbauer GmbH, Singen, Germany) operating at 50 kHz and maintained at 45 °C for 20 min. Following extraction, the mixtures were centrifuged at 4000 rpm for 10 min, and the supernatants were filtered through Whatman No. 1 filter paper to remove residual plant material. The ethanolic and hydroethanolic filtrates were concentrated under reduced pressure using a BUCHI R-300 rotary evaporator (BÜCHI Labortechnik AG, Flawil, Switzerland). For the aqueous extract, most of the water was first removed under reduced pressure using the same rotary evaporator, after which the concentrated aqueous extract was lyophilized to obtain a dry crude extract. The resulting crude extracts were weighed and stored at 4 °C until further analysis. The extraction yield was calculated relative to the initial mass of dry powdered plant material according to Equation (1):

Extraction yield (%) = (mass of dried crude extract/initial mass of dry powdered plant material) × 100(1)

#### 4.1.3. Chemicals and Reagents

All chemicals and reagents used in this study were of analytical grade unless otherwise specified. Folin–Ciocalteu reagent, gallic acid, quercetin, anthrone, bovine serum albumin (BSA), Bradford reagent, 2,2-diphenyl-1-picrylhydrazyl (DPPH), α-amylase, soluble starch, 3,5-dinitrosalicylic acid (DNS), mushroom tyrosinase, L-3,4-dihydroxyphenylalanine (L-DOPA), kojic acid, acarbose, crystal violet, and azithromycin were purchased from Sigma-Aldrich (St. Louis, MO, USA). Unless otherwise specified, aqueous solutions used in the phytochemical and biological assays were prepared using distilled water.

### 4.2. Phytochemical Analysis

#### 4.2.1. Determination of Total Phenolic Content (TPC)

Total phenolic content (TPC) was evaluated using a microplate-based Folin–Ciocalteu assay adapted from the procedure reported in [54]. For each determination, 20 µL of extract solution (1 mg mL^−1^) was combined with 100 µL of Folin–Ciocalteu reagent (10%, *v*/*v*) in a 96-well microplate. The reaction was allowed to proceed for 5 min at room temperature under light-protected conditions before adding 80 µL of sodium carbonate (7.5%, *w*/*v*). The plate was then maintained for 30 min at room temperature, and the absorbance was recorded at 765 nm using a microplate reader (FLUOstar Omega, BMG LABTECH, Ortenberg, Germany). Quantification was based on a gallic acid calibration curve, and the results were expressed as milligrams of gallic acid equivalents per gram of dry extract (mg GAE g^−1^ extract).

#### 4.2.2. Determination of Total Flavonoid Content (TFC)

Total flavonoid content (TFC) was assessed by formation of flavonoid–aluminum complexes using the aluminum chloride colorimetric method, with quercetin as the calibration standard [55]. An aliquot of each extract solution (100 µL, 1 mg mL^−1^) was combined with aluminum chloride (1.2%) and potassium acetate (120 mM). The reaction mixtures were maintained at room temperature for 30 min to allow color development. Absorbance was subsequently recorded at 415 nm against the corresponding reagent blank using a microplate reader (BMG LABTECH, Ortenberg, Germany). TFC was calculated from the quercetin calibration curve and reported as milligrams of quercetin equivalents per gram of dry extract (mg QE g^−1^ extract). Three separately prepared aliquots of each extract were analyzed (*n* = 3).

#### 4.2.3. Determination of Total Sugar Content (TSC)

Total sugar content (TSC) was determined using the anthrone colorimetric method described in [56]. An aliquot (100 µL) of each extract was combined with 900 µL of freshly prepared anthrone reagent. The mixtures were heated at 100 °C for 7 min and subsequently allowed to cool to room temperature. A 200 µL aliquot of each reaction mixture was then transferred to a 96-well microplate, and absorbance was recorded at 620 nm against the corresponding reagent blank using a microplate reader (BMG LABTECH, Ortenberg, Germany). Glucose was used to establish the calibration curve, and TSC was expressed as milligrams of glucose equivalents per gram of dry extract (mg GE g^−1^ extract). Three separately prepared aliquots of each extract were analyzed (*n* = 3).

#### 4.2.4. Determination of Total Protein Content (TP)

Total protein content (TP) was quantified using the Bradford colorimetric assay according to the method described in [57]. For each determination, 40 µL of extract solution (1 mg mL^−1^) was dispensed into a 96-well microplate and mixed with 160 µL of Bradford reagent. After allowing the reaction to develop for 5 min at room temperature, absorbance was recorded at 595 nm against the corresponding reagent blank using a microplate reader (BMG LABTECH, Ortenberg, Germany). Protein content was calculated from a bovine serum albumin (BSA) calibration curve and expressed as milligrams of bovine serum albumin equivalents per gram of dry extract (mg BSAE g^−1^ extract). Three separately prepared aliquots of each extract were analyzed (*n* = 3).

#### 4.2.5. LC-ESI-MS/MS Analysis

The phytochemical composition of the extracts was characterized using a Shimadzu LCMS-8050 liquid chromatography–tandem mass spectrometry system (Shimadzu, Kyoto, Japan) equipped with a triple quadrupole mass analyzer and an electrospray ionization (ESI) source. Chromatographic separation was performed on a Zorbax Eclipse XDB-C18 reversed-phase column (4.6 × 150 mm, 3.5 µm; Agilent Technologies, Santa Clara, CA, USA). The mobile phase consisted of water (solvent A) and acetonitrile (solvent B), both containing 0.1% formic acid. A linear gradient was applied from 5% to 90% solvent B over 60 min at a flow rate of 1.0 mL min^−1^. Samples (10 µL, 10 mg mL^−1^) were introduced using a SIL-40C XS autosampler (Shimadzu Corporation, Kyoto, Japan), and instrument operation and data acquisition were managed with LCsolution software (version 1.25, Shimadzu, Kyoto, Japan). Mass spectrometric analyses were performed in the negative ionization mode, with spectra acquired over an *m*/*z* range of 100–1500. MS/MS fragmentation experiments were conducted using collision energies of 35 and 45 eV. Compounds were tentatively annotated based on their retention time, molecular ions, and MS/MS fragmentation patterns through comparison with published literature and an in-house database. Citric acid, gallic acid, chlorogenic acid, esculetin, and caffeic acid were additionally confirmed by comparison with in-house reference standards. For compounds not confirmed using reference standards, LC–MS/MS data did not necessarily permit unequivocal differentiation of structural or positional isomers, exact sugar attachment positions, or anomeric configurations [58].

### 4.3. Evaluation of Antioxidant Activity

#### 4.3.1. DPPH Radical Scavenging Assay

The radical-scavenging activity of the *R. pentaphylla* extracts was determined using the DPPH assay according to the method described in [59]. A series of extract solutions covering concentrations from 0.001 to 1 mg mL^−1^ was prepared. For each concentration, 20 µL of the extract solution was combined with 180 µL of freshly prepared methanolic DPPH solution (0.1 mM) in a 96-well microplate. The reaction mixtures were maintained at room temperature in the dark for 30 min, after which absorbance was recorded at 517 nm using a microplate reader (BMG LABTECH, Ortenberg, Germany). Methanol was used as the blank, while ascorbic acid served as the positive control. DPPH radical-scavenging activity was calculated according to Equation (2):

DPPH inhibition (%) = [(A_control_ − A_sample_)/A_control_] × 100 (2)

where A_control_ and A_sample_ represent the absorbance of the control and the extract-containing reaction mixture, respectively. The IC_50_ value, defined as the extract concentration required to achieve 50% DPPH radical inhibition, was obtained from the corresponding concentration–response curve. Three separately prepared aliquots of each extract were analyzed (*n* = 3). A blank containing the DPPH solution and methanol instead of the extract was prepared, while ascorbic acid was used as the positive control.

#### 4.3.2. Ferric-Reducing Capacity Assay

The ferric-reducing capacity of the *R. pentaphylla* extracts was evaluated using a microplate-based ferricyanide reducing assay according to the method reported in [60]. For each determination, 30 µL of extract was introduced into a 96-well microplate and sequentially mixed with 100 µL of distilled water, 45 µL of HCl (1 M), 45 µL of potassium ferricyanide (1%, *w*/*v*), 15 µL of sodium dodecyl sulfate (SDS; 0.2%, *w*/*v*), and 15 µL of FeCl_3_ (1%, *w*/*v*). The reaction mixtures were incubated at 50 °C for 20 min, after which absorbance was recorded at 700 nm using a microplate reader (BMG LABTECH, Ortenberg, Germany). Quantification was performed using a FeSO_4_ calibration curve, and the results were expressed as mM FeSO_4_ equivalents g^−1^ extract. Three separately prepared aliquots of each extract were analyzed (*n* = 3).

### 4.4. Enzymatic Activity

#### 4.4.1. α-Amylase Inhibition

The α-amylase inhibitory activity of the *R. pentaphylla* extracts was evaluated according to the method described in [61], with modifications. For each assay, 100 µL of extract solution was mixed with 100 µL of α-amylase solution (1 U mL^−1^) prepared in phosphate-buffered saline (PBS). The mixtures were pre-incubated at 37 °C for 10 min, after which 100 µL of soluble starch solution (1%, *w*/*v*) was added as the substrate. The reaction was allowed to proceed at 37 °C for an additional 30 min and was subsequently terminated by adding 200 µL of 3,5-dinitrosalicylic acid (DNS) reagent. The mixtures were then heated in a boiling water bath for 10 min. After cooling to room temperature, 1.5 mL of distilled water was added, and absorbance was measured at 540 nm using a UV–Vis spectrophotometer (BMG LABTECH, Ortenberg, Germany).

For each extract concentration, a corresponding sample blank containing the extract and all assay reagents except the enzyme was prepared to correct for the intrinsic absorbance of the extract. The absorbance of the sample blank was subtracted from that of the corresponding reaction mixture, and the resulting corrected absorbance values were used to calculate α-amylase inhibition.

Acarbose was used as the positive control. α-Amylase inhibition was calculated according to Equation (3):

α-Amylase inhibition (%) = [(A_control_ − A_sample_)/A_control_] × 100 (3)

where A_control_ represents the absorbance of the control corresponding to 100% enzyme activity, and A_sample_ represents the blank-corrected absorbance of the reaction mixture containing the extract. Three separately prepared aliquots of each extract were analyzed (*n* = 3).

#### 4.4.2. Tyrosinase Inhibition

Tyrosinase inhibitory activity was evaluated using mushroom tyrosinase and L-3,4-dihydroxyphenylalanine (L-DOPA) as the substrate, according to the method described in [62]. Each reaction mixture contained 20 µL of extract, 20 µL of mushroom tyrosinase solution (2500 U mL^−1^), and 100 µL of phosphate buffer (0.05 M, pH 6.5). The enzymatic reaction was initiated by the addition of 20 µL of L-DOPA solution (5 mM). Following incubation for 20 min, dopachrome formation was monitored by recording the absorbance at 475 nm using a microplate reader (BMG LABTECH, Ortenberg, Germany).

For each extract concentration, a corresponding sample blank containing the extract and all assay components except tyrosinase was included to correct for the intrinsic absorbance of the extract at 475 nm. The absorbance of the sample blank was subtracted from that of the corresponding reaction mixture, and the resulting corrected absorbance values were used to calculate tyrosinase inhibition.

Kojic acid was used as the reference inhibitor. Tyrosinase inhibition was calculated according to Equation (4):

Tyrosinase inhibition (%) = [(A_control_ − A_sample_)/A_control_] × 100 (4)

where A_control_ represents the absorbance of the control corresponding to 100% enzyme activity, and A_sample_ represents the blank-corrected absorbance of the reaction mixture containing the extract. Three separately prepared aliquots of each extract were analyzed (*n* = 3).

### 4.5. Antibacterial and Antibiofilm Assays

#### 4.5.1. Disc Diffusion Test

The antibacterial activity of the *R. pentaphylla* extracts was evaluated using the agar disc diffusion method against five bacterial strains: *P. aeruginosa*, *E. coli*, *E. faecalis*, *S. aureus*, and *S. typhi*. All bacterial strains were clinical pathogenic isolates kindly provided by the Microbiology Laboratory of the Faculty of Medicine and Pharmacy of Marrakech (FMPM), Morocco. An aliquot (100 µL) of each overnight bacterial culture was uniformly spread onto Mueller–Hinton agar plates. Sterile paper discs (6 mm in diameter) were loaded with 30 µL of extract solution (100 mg mL^−1^) and placed on the inoculated agar surface. The plates were incubated at 37 ± 2 °C for 24 h. Ampicillin (40 µg mL^−1^) was used as the reference antibacterial agent, whereas the corresponding extraction solvents served as negative controls. Antibacterial activity was assessed by measuring the diameter of the inhibition zone around each disc, and the results were expressed in millimetres (mm) [63]. All assays were performed in triplicate.

#### 4.5.2. MIC Determination for Extracts

Based on the preliminary agar disc diffusion screening, only the ethanolic (EtOH_100_) and hydroethanolic (EtOH_50_) extracts of *R. pentaphylla* were selected for MIC determination against *P. aeruginosa*, whereas the aqueous extract was not further evaluated because of its weak inhibitory effect in the disc diffusion assay. The MIC was determined using the broth microdilution method in 96-well microplates according to previously described procedures [60,63,64]. Due to extract solubility limitations in the growth medium, 100 mg mL^−1^ was the highest concentration tested. Two-fold serial dilutions of the extracts were prepared in Mueller–Hinton broth to obtain final concentrations ranging from 100 to 1.562 mg mL^−1^. Before testing, the extract solutions were sterilized by filtration through 0.22 µm syringe filters.

Aliquots (200 µL) of each extract dilution were dispensed into the microplate wells and inoculated with 2 µL of a bacterial suspension adjusted to an OD_600_ of 0.6. Wells containing the bacterial inoculum without extract served as growth controls, whereas uninoculated Mueller–Hinton broth was used as a sterility control. Following incubation at 37 °C for 18 h with shaking at 150 rpm, bacterial growth was assessed both visually and spectrophotometrically by measuring the absorbance at 600 nm. The MIC was defined on the basis of the visual endpoint as the lowest tested extract concentration showing no visible bacterial growth. Spectrophotometric measurements were used separately to quantify bacterial growth inhibition. When complete inhibition of visible bacterial growth was not achieved at the highest concentration tested, the MIC was reported as >100 mg mL^−1^.

#### 4.5.3. Biofilm Inhibition Assay

The effect of *R. pentaphylla* extracts on biofilm formation by *P. aeruginosa* was evaluated using the crystal violet microplate assay according to previously described methods [65,66]. Based on the antibacterial screening, only the ethanolic (EtOH_100_) and hydroethanolic (EtOH_50_) extracts were selected for further evaluation. Both extracts were tested at concentrations of 12.5 and 25 mg mL^−1^. Sterile extract solutions were prepared in Mueller–Hinton broth (MHB), inoculated with a standardized bacterial suspension, and incubated under the same conditions as those used for the MIC assay.

Following incubation, non-adherent cells were removed, and the remaining adherent biofilm was stained with 1% crystal violet for 15 min. The wells were subsequently washed to remove excess stain and allowed to air-dry. The retained crystal violet was then solubilized with 95% ethanol, and biofilm biomass was quantified by measuring absorbance at 595 nm using a microplate reader (BMG LABTECH, Ortenberg, Germany). Wells containing inoculated medium without extract served as the untreated growth control, whereas uninoculated medium served as the sterility control. Azithromycin (2 µg mL^−1^) was included as the reference antibiofilm agent. All experiments were performed in triplicate.

#### 4.5.4. Total Extracellular Protein Quantification

The effect of *R. pentaphylla* extracts on the total extracellular protein content of *Pseudomonas aeruginosa* cultures was evaluated using the Bradford colorimetric assay according to previously described methods [66,67,68]. Based on the antibacterial screening, only the ethanolic (EtOH_100_) and hydroethanolic (EtOH_50_) extracts were selected for further evaluation. Both extracts were tested at concentrations of 12.5 and 25 mg mL^−1^. Bacterial cultures were incubated in the presence or absence of the extracts for 24 h under the same experimental conditions used for the MIC assay. Following incubation, the cultures were centrifuged at 10,000× *g* for 12 min, and the resulting supernatants were filtered through 0.45 µm syringe filters. Total extracellular protein content in the resulting cell-free supernatants was determined using the Bradford assay, and absorbance was measured at 595 nm. Azithromycin (2 µg mL^−1^) was included as the reference control, whereas uninoculated culture medium served as the blank.

#### 4.5.5. Exopolysaccharide Estimation

In addition to total extracellular protein quantification, extracellular polymeric substances (EPS) were recovered from the collected cell-free supernatants by precipitation with cold 95% ethanol followed by overnight incubation at 4 °C. The resulting EPS pellets were subsequently used to determine their total carbohydrate content according to established methods [68]. Azithromycin (2 µg mL^−1^) was included as the reference control, while uninoculated culture medium served as the blank.

### 4.6. Statistical Analysis

Data are expressed as the mean ± standard deviation (SD). Statistical analyses were performed using GraphPad Prism version 8.0.1 (GraphPad Software, San Diego, CA, USA), and statistical significance was set at *p* < 0.05. Data normality and homogeneity of variances were assessed using the Shapiro–Wilk and Brown–Forsythe tests, respectively.

For phytochemical and chemical assays, *n* = 3 corresponded to three replicate measurements performed using separately prepared aliquots of the same extract and did not represent three independent extractions. For biological assays, including antibacterial, biofilm, extracellular protein, and EPS-associated carbohydrate assays, *n* = 3 represented three independent biological experiments performed using independently prepared bacterial cultures. When technical replicates were included within an independent biological experiment, their mean was considered the experimental unit for statistical analysis.

Statistical tests were selected according to the experimental design. One-way analysis of variance (ANOVA), followed by Tukey’s multiple-comparisons test, was used for experiments involving a single factor. For experiments involving both extract type and treatment concentration, two-way ANOVA followed by the appropriate multiple-comparisons test was used. Exact adjusted *p*-values and relevant statistical information are reported in the corresponding Results sections and figure legends, where applicable.

## 5. Conclusions

This study provides an integrated evaluation of the phytochemical profiles and in vitro biological properties of Moroccan *R. pentaphylla* extracts obtained using solvents of different polarities. LC–ESI–MS/MS profiling resulted in the annotation of 39 metabolites belonging to several phytochemical classes, including phenolic acids, flavan-3-ols, coumarins, and flavonoid glycosides. Citric acid, gallic acid, chlorogenic acid, esculetin, and caffeic acid were confirmed by comparison with in-house reference standards, whereas the remaining compounds were tentatively annotated based on their precursor ions and MS/MS fragmentation patterns through comparison with published literature and an in-house database. Differences in the relative chromatographic responses of individual compounds among the aqueous, hydroethanolic, and ethanolic extracts highlighted the influence of extraction solvent on the resulting phytochemical profiles.

The biological responses also varied according to solvent composition. EtOH_50_ exhibited the highest total phenolic content and the strongest activity in the DPPH and ferric-reducing capacity assays, whereas the aqueous and EtOH_100_ extracts showed greater tyrosinase inhibitory activity than EtOH_50_. The three extracts exhibited comparable α-amylase inhibitory activity, with no statistically significant differences among them. Antibacterial activity was generally weak or limited. Nevertheless, EtOH_100_ produced a comparatively greater effect against *Pseudomonas aeruginosa* in the disc-diffusion assay. Broth microdilution indicated an MIC of 100 mg mL^−1^ for EtOH_100_, whereas the MIC of EtOH_50_ was not reached within the tested concentration range and was therefore reported as >100 mg mL^−1^.

At the tested concentrations of 12.5 and 25 mg mL^−1^, EtOH_100_ significantly reduced *P. aeruginosa* biofilm biomass, whereas EtOH_50_ produced a significant reduction only at 25 mg mL^−1^. Both extracts also significantly reduced the measured extracellular protein levels. By contrast, their effects on EPS-associated carbohydrates were more limited and did not reach statistical significance in the individual comparisons with the untreated control. These findings indicate that the biological response profiles of *R. pentaphylla* extracts vary according to solvent composition and that no single extract showed the same pattern of activity across all evaluated endpoints.

Although five compounds were confirmed using in-house reference standards, the remaining LC–ESI–MS/MS assignments are tentative, and no individual metabolite was isolated, quantified using a compound-specific calibration curve, or evaluated independently. Consequently, direct causal relationships between particular metabolites and the observed biological effects cannot be established from the present data. Moreover, the biofilm-associated findings should be interpreted cautiously because the biofilm and extracellular matrix measurements were not normalized to bacterial biomass or viable cell counts.

Bioassay-guided fractionation, followed by the isolation, structural confirmation, compound-specific quantification, and individual biological evaluation of candidate metabolites, will be required to clarify the contribution of specific constituents. Combination-based testing would also help determine whether additive, synergistic, or antagonistic interactions contribute to the activities of these chemically complex extracts. Further mechanistic, safety, and in vivo investigations will be necessary to assess the biological relevance and potential therapeutic or cosmeceutical applications of *R. pentaphylla* extracts.

## Figures and Tables

**Table 1:** Extraction yield and phytochemicals of *R. pentaphylla*.

Extract	Yield	TPC	TFC	TP	TS
	%	mg GAE-eq g^−1^	mg QE-eq g^−1^	mg BSAE-eq g^−1^	mg GLU-eq g^−1^
Aqueous	6.77	116.69 ± 0.01 ^c^	36.94 ± 0.01 ^c^	15.99 ± 0.02 ^c^	144.82 ± 0.02 ^a^
EtOH_50_	37.95	161.05 ± 0.01 ^a^	51.34 ± 0.01 ^b^	38.02 ± 0.01 ^b^	84.40 ± 0.01 ^b^
EtOH_100_	24.5	142.99 ± 0.01 ^b^	66.50 ± 0.01 ^a^	90.19 ± 0.01 ^a^	135.48 ± 0.03 ^a^

Values are expressed as the mean ± SD (*n* = 3). According to one-way ANOVA followed by Tukey’s multiple comparisons test (*p* < 0.05), means sharing different superscript letters (a, b, c) within the same column are significantly different.

**Table 2:** Phytochemical profile of *Rhus pentaphylla* extracts tentatively annotated by LC–ESI–MS/MS.

Rt (min)	[M − H]^−^	MS/MS	Proposed Component	ETOH	ETOH_50_	Aqueous
0.91	193	193	Glucuronic acid	Tr	Tr	Tr
1.47	191	111, 191	Citric acid *	+	+	+
1.53	191	108, 191	Quinic acid	+	+	+
1.61	353	173, 191	Quinic hexoside	++	+++	+++
2.80	331	169, 331	Galloyl hexoside	+	Tr	+
3.07	609	177, 305	(epi)Gallocatechin-(epi) gallocatechin	+	Tr	+
3.25	169	125	Gallic acid *	+	Tr	+
3.56	315	109, 153	Protocatechuic acid glucoside	+	Tr	+
5.22	305	125, 179, 305	(epi)Gallocatechin	+	Tr	+
5.49	593	177, 289, 305	(epi) Gallocatechin-(epi)catechin	+	+	+
6.32	353	135, 179, 191	Chlorogenic acid *	+++	++++	++++
6.90	341	135	Caffeic acid glucoside	+	+	+
8.27	337	119, 191	*p*-Coumaroylquinic acid	+	+	+
9.22	325	119, 145	Coumaric acid glucoside	+++	+	+
9.48	357	137, 195	Dihydroferulic acid glucoside	+++	+	+
9.85	353	135, 179, 191	Cryptochlorogenic acid	+++	++++	++++
10.32	177	109	Esculetin *	+++	++	++
10.51	457	119, 163	Coumaric acid glucoside arabinoside	+++	++	++
10.78	179	135	caffeic acid *	+	+	+
12.23	289	109, 158	Catechin	+	+	+
14.47	163	119, 163	Coumaric acid	+++	++	++
14.56	625	271, 317	Myricetin rutinoside	+++	++	++
14.75	625	301	Quercetin diglucoside	+++	++	++
15.29	479	271, 317	Myricetin Glucoside	+++	++	+
15.53	493	317	Myricetin Glucuronide	+++	++	+
15.58	463	301	Quercetin Glucoside	+++	+++	+
16.39	449	271, 317	Myricetin arabinoside	+++	++	+
17.38	609	301	Quercetin rutinoside (Rutin)	+++	+++	++
18.32	477	301	Quercetin Glucuronide	++++	+++	+++
19.20	435	301	Quercetin Arabinoside	++	++	+
19.98	593	285	Kaempferol rutinoside	++	++	+
20.49	623	301, 315	Isorhamnetin rutinoside (Narcissin)	++++	++++	+++
21.39	477	301, 315	Isorhamnetin glucoside	++++	++++	++
22.95	447	285	Kaempferol glucoside	+++	+++	++
23.79	431	285	Kaempferol Rhamnoside	++	++	++
27.96	301	151, 179	Quercetin	+	+	+
27.97	317	163, 301, 317	Myricetin	+++	++	++
33.04	285	255	Kaempferol	++	++	Tr
33.90	315	151, 271, 301	Isorhamnitin	++	++	Tr

* Compounds confirmed by comparison with in-house reference standards. All other compounds were tentatively annotated based on molecular ions, MS/MS fragmentation patterns, literature data, and comparison with an in-house database. Tr, +, ++, +++, and ++++ indicate increasing relative chromatographic responses based on comparison of the peak areas (AUCs) of the corresponding compound among the three extracts. These symbols provide a semi-quantitative comparison only and do not represent absolute concentrations, as compound-specific calibration curves were not established.

**Table 3:** Antioxidant activity of *R. pentaphylla* extracts.

Extract	DPPH IC_50_, µg/mL	Ferric-Reducing Capacity mM FeSO_4_/g
Aqueous	87.01 ± 0.01 ^a^	66.57 ± 0.01 ^b^
EtOH_50_	32 ± 0.01 ^c^	81.56 ± 0.07 ^a^
EtOH_100_	39.60 ± 0.02 ^b^	70.44 ± 0.06 ^ab^
Ascorbic acid	3.60 ± 0.01	–
Quercetin	–	57.48 ± 0.55

Values are expressed as the mean ± SD. Different letters within the same column indicate significant differences (*p* < 0.05; one-way ANOVA followed by Tukey’s test).

**Table 4:** α-amylase and tyrosinase inhibitory activities of *R. pentaphylla* extracts expressed as IC_50_ values (mg/mL).

Extract	α-Amylase IC_50_ (mg/mL)	Tyrosinase IC_50_ (mg/mL)
Aqueous	1.25 ± 0.08 ^a^	0.27 ± 0.01 ^b^
EtOH_50_	1.49 ± 0.09 ^a^	0.42 ± 0.04 ^a^
EtOH_100_	1.60 ± 0.12 ^a^	0.27 ± 0.05 ^b^
Acarbose	0.61 ± 0.03	–
Kojic acid	–	0.06 ± 0.05

Values are expressed as the mean ± SD (*n* = 3). Different superscript letters within the same column indicate statistically significant differences among *R. pentaphylla* extracts as determined by one-way ANOVA followed by Tukey’s multiple comparisons test (*p* < 0.05). Lower IC_50_ values indicate stronger enzyme inhibitory activity.

**Table 5:** Antibacterial activity of *R. pentaphylla* extracts against the tested bacterial strains expressed as inhibition zone diameters (mm).

	Diameters of the Inhibition Zones (mm)			
	EtOH_100_	Aqueous Extract	EtOH_50_	Ampicillin
*P. aeruginosa*	14.01 ± 0.62 ^a^	6.82 ± 0.05 ^b^	7.44 ± 0.01 ^b^	15.01 ± 0.03
*E. coli*	6.47 ± 0.03 ^a^	6.48 ± 0.06 ^a^	6.52 ± 0.03 ^a^	14.71 ± 0.01
*E. faecalis*	6.31 ± 0.01 ^a^	6.37 ± 0.01 ^a^	6.43 ± 0.08 ^a^	15.81 ± 0.02
*S. aureus*	6.44 ± 0.09 ^a^	6.39 ± 0.02 ^a^	6.31 ± 0.02 ^a^	17.22 ± 0.04
*S. typhi*	6.39 ± 0.06 ^a^	6.30 ± 0.01 ^a^	6.47 ± 0.05 ^a^	16.45 ± 0.06

Values are expressed as the mean ± SD (*n* = 3). Different superscript letters (a, b) within the same row indicate statistically significant differences among the *R. pentaphylla* extracts, as determined by one-way ANOVA followed by Tukey’s multiple-comparisons test (*p* < 0.05). Inhibition zone diameters include the 6 mm paper disc.

**Table 6:** Minimum inhibitory concentration (MIC) and bacterial growth inhibition of *R. pentaphylla* extracts against *P. aeruginosa*.

Extract	MIC (mg mL^−1^)	Growth Inhibition at 100 mg mL^−1^ (%)
EtOH_50_	>100	81.56 ± 0.07
EtOH_100_	100	98.25 ± 0.01
Ampicillin	0.031	ND

ND, not determined. The MIC was defined as the lowest tested concentration showing no visible bacterial growth. Due to extract solubility limitations, 100 mg mL^−1^ was the highest concentration tested. Growth inhibition percentages were determined spectrophotometrically at 100 mg mL^−1^ and are reported separately from the visual MIC endpoint.

## Data Availability

The original contributions presented in this study are included in the article. Further inquiries can be directed to the corresponding authors.
